# Global Longitudinal Strain Accuracy for Cardiotoxicity Prediction in a Cohort of Breast Cancer Patients During Anthracycline and/or Trastuzumab Treatment

**DOI:** 10.5935/abc.20180021

**Published:** 2018-02

**Authors:** Eliza de Almeida Gripp, Gabriela Escudini de Oliveira, Luiz Augusto Feijó, Marcelo Iorio Garcia, Sergio Salles Xavier, Andréa Silvestre de Sousa

**Affiliations:** Hospital Universitário Clementino Fraga Filho - Universidade Federal do Rio de Janeiro (UFRJ), Rio de Janeiro, RJ - Brazil

**Keywords:** Breast Neoplasms/drug therapy, Cardiotoxicity, Stroke Volume, Trastuzumab, Indicators of Morbidity and Mortality

## Abstract

**Background:**

The high cardiotoxicity morbidity and mortality rates associated with the antineoplastic therapy for breast cancer could be reduced with the early use of cardioprotective drugs. However, the low sensitivity of left ventricular ejection fraction limits its use in that preventive strategy. New parameters, such as global longitudinal strain, are being used in the early detection of contractile function changes.

**Objectives:**

To assess the incidence of cardiotoxicity in patients treated for breast cancer, the independent factors associated with that event, and the ability of strain to identify it early.

**Methods:**

Prospective observational study of consecutive outpatients diagnosed with breast cancer, with no previous antineoplastic treatment and no ventricular dysfunction, who underwent anthracycline and/or trastuzumab therapy. The patients were quarterly evaluated on a 6- to 12-month follow-up by an observer blind to therapy. Cox regression was used to evaluate the association of cardiotoxicity with clinical, therapeutic and echocardiographic variables. A ROC curve was built to identify the strain cutoff point on the third month that could predict the ejection fraction reduction on the sixth month. For all tests, the statistical significance level adopted was p ≤ 0.05.

**Results:**

Of 49 women (mean age, 49.7 ± 12.2 years), cardiotoxicity was identified in 5 (10%) on the third (n = 2) and sixth (n = 3) months of follow-up. Strain was independently associated with the event (p = 0.004; HR = 2.77; 95%CI: 1.39-5.54), with a cutoff point for absolute value of -16.6 (AUC = 0.95; 95%CI: 0.87-1.0) or a cutoff point for percentage reduction of 14% (AUC = 0.97; 95%CI: 0.9-1.0).

**Conclusion:**

The 14% reduction in strain (absolute value of -16.6) allowed the early identification of patients who could develop anthracycline and/or trastuzumab-induced cardiotoxicity.

## Introduction

Advances in the treatment of several tumors, such as the new antineoplastic drugs, have improved the survival of patients with cancer, resulting in more than 12 million survivors.^1^ That, however, has allowed the identification of side effects, such as cardiotoxicity, responsible for an increase in mortality.^2,3^

In 2016, the European Society of Cardiology has published a position paper recommending the diagnosis of cardiotoxicity be made in the presence of an ejection fraction (EF) reduction >10% for values below normality (53%).^4^ Prior to that publication, different definitions of cardiotoxicity were used, hindering the assessment of its real incidence.^2^ The most commonly used definition has been elaborated by the committee of cardiac review and assessment of trastuzumab-related cardiotoxicity, and consists of a reduction of 5% or more in EF values lower than 55%, accompanied by signs and/or symptoms of heart failure (HF), or a reduction of 10% or more in EF values lower than 55%, without clinical findings of HF.^5-8^

Cardiotoxicity is a well-established side effect of several antineoplastic drugs, particularly anthracyclines and trastuzumab, used for breast cancer treatment.^9,10^

The identification of patients at high risk for developing cardiotoxicity would be the ideal strategy to reduce mortality.

Global longitudinal strain (GLS) is used in clinical practice aimed at the early detection of changes in myocardial contractile function.^11^ However, neither GLS use nor its cutoff point to predict cardiotoxicity have been standardized.

The American Society of Echocardiography and the European Association of Cardiovascular Imaging have agreed that deformity changes precede ventricular dysfunction. A reduction > 15% in GLS, immediately after or during anthracycline treatment, was the most useful parameter to predict cardiotoxicity, while a reduction < 8% might exclude its diagnosis.^12^ However, there is a grey zone between those values.

This study was aimed at assessing the incidence of breast cancer treatment-induced cardiotoxicity, identifying the independent risk factors associated with that event (drugs, dose, radiotherapy, clinical data and echocardiographic variables), and at identifying the best GLS cutoff point for the early detection of cardiotoxicity, prior to EF reduction.

## Methods

This is a prospective and observational study of consecutive patients referred to the Oncology Outpatient Clinic of the Clementino Fraga Filho University-Affiliated Hospital (HUCFF), Rio de Janeiro, Brazil, with confirmed diagnosis of breast cancer and indication for potentially cardiotoxic antineoplastic treatment. Data were collected from January 22, 2015, to June 19, 2016, by filling in a form consisting of patient's clinical information, physical exam, echocardiographic data and proposed treatment.

The inclusion criteria were: age ≥ 18 years; diagnosis of breast cancer, with neither previous antineoplastic treatment nor radiotherapy; normal EF, according to the last recommendations of the American Society of Echocardiography and the European Association of Cardiovascular Imaging^13^ (> 54%, by use of the Simpson's method), on the first Doppler echocardiogram before treatment; and antineoplastic treatment planning with anthracyclines and/or trastuzumab.

The exclusion criteria were as follows: impossibility of accurately assessing GLS because of an inappropriate acoustic window; presence of cardiac arrhythmias and/or non-sinus rhythms; use of beta-blockers and/or angiotensin-converting-enzyme inhibitors and/or angiotensin receptor blockers; and moderate or severe heart valve disease.

The patients who met the inclusion criteria underwent Doppler echocardiography at baseline, before initiating the anthracycline, and then every 3 months, during a 6- to 12-month follow-up at the HUCFF. All tests were performed by one single professional, who was blind to the treatment instituted. Two distinct protocols of antineoplastic drugs were used:

FEC (5-fluorouracil 500 mg/m^2^, epirubicin 100 mg/m^2^ and cyclophosphamide 500 mg/m^2^) in 3 cycles, every 21 days, followed by docetaxel 100 mg/m^2^ in other 3 cycles, every 21 days;
2. Doxorubicin 60 mg/m^2^ and cyclophosphamide 600 mg/m^2^ in 4 cycles, every 21 days, followed by paclitaxel 80 mg/m^2^ weekly, for 12 cycles, for both adjuvant and neoadjuvant treatments.

The patients eligible for trastuzumab should undergo genetic assessment with human epidermal growth factor receptor 2 (HER2) test. Those who had a positive HER2 test result (+++/+++) or undetermined HER2 test result (++/+++), but positive FISH (*Fluorescence in Situ Hybridization*), were assigned to adjuvant treatment. Trastuzumab would be offered for 1 year, with 18 applications at 21-day intervals, with an initial dose of 8 mg/kg, followed by a maintenance dose of 6mg/m^2^.

In 19 months (01/22/2015 to 06/19/2016), 58 patients were referred to the Oncology Service of the HUCFF to undergo Doppler echocardiography. Of those 58 patients, 9 were excluded because of inappropriate acoustic window (2 were on beta-blockers), leaving 49 patients as the study population.

### Doppler echocardiography

Doppler echocardiography was performed with the patient at rest in the left lateral position, using the Vivid S6-GE device (GE, Vingmed Ultrassound Horten, Norway), LCD 17" monitor, with image acquisition with a 3S transducer and harmonic imaging. The measurements were reassessed by a second observer, also blind to the treatment instituted and specialized in the method. Interobserver agreement was assessed. All tests were performed with the same device. Sector and depth were adjusted to optimize the image. The measurements and image acquisition followed the recommendations of the American Society of Echocardiography and the European Association of Cardiovascular Imaging.^13^

The following echocardiographic variables were assessed: EF, calculated by use of the Simpson's method, considering the normal value of EF > 54% for the female sex, according to the current recommendations;^13^ diastolic function, evaluated by use of mitral flow with anterograde values of E wave and A wave, tissue Doppler of septal and lateral mitral annulus, measures of S' wave (systolic velocity of the mitral ring) and E/E' ratio; S wave of the right ventricle (cm/s); indexed left atrial volume (mL/m^2^); tricuspid annular plane systolic excursion (TAPSE); and pulmonary artery systolic pressure (PASP).

In our study, cardiotoxicity was defined, in accordance with the cardiac review committee and expert recommendations on trastuzumab-related cardiotoxicity, as a reduction of at least 5% of EF values < 55% in symptomatic patients, or a reduction of at least 10% of EF values < 55% in asymptomatic patients.^8^

The GLS was acquired by use of Automated Functional Imaging (AFI) of three clips with images of the left ventricle on three apical views, so that all myocardial segments could be well visualized: 4-chamber, 2-chamber and 3-chamber views. The events of aortic valve opening and closure were marked. The images were acquired at a frame rate of 40-90 fps (> 70% of heart rate). Right ventricular (RV) strain was acquired by use of AFI. The acquisition of a clip of apical window projection adapted to RV assessment was necessary to include the entire RV free wall and its tip for further analysis. Three points were marked in the basal segments (inferior septum, tricuspid annulus) and apex. After that marking, the analysis was performed in the same way described for the left ventricle.

The images were analyzed in the same device and same working station (EchoPAC 13.0, GE Vingmed Ultrassound Horten, Norway).

### Reproducibility

The measures of left ventricular (LV) GLS, RV strain and RV free wall strain underwent intra- and interobserver agreement analysis by use of intraclass correlation coefficients. Bland-Altman plots were created to show the results of the interobserver analyses.

Tests at different follow-up times were randomly drawn, defining a sample of approximately 10% of all calculations of the GLS analyzed during the study. Data were reassessed by the same observer, blind to the treatment instituted and specialized in the method, so that intraobserver agreement could be assessed.

The interobserver analysis was performed by another professional, also specialized in the method with experience in GLS assessment, the major variable of this study. The second observer used the same clip selected by the first observer, with predefined configurations, such as depth, gain, value of pulse repetition frequency (PRF); however, the new regions of interest for myocardial markers were freely chosen during the reanalysis. If the observer agreed on the region of interest marked, the next step would be the approval of the six segments according to the walls assessed. Upon approval with a command on the working station screen, the values of GLS and segment strains were calculated and demonstrated by use of bull's eye. Therefore, the calculations of the LV GLS, RV strain and RV free wall strain were repeated at the working station by the second observer, who was blind to the time the Doppler echocardiography was performed, the treatment and the patient's outcome.

### Statistical analysis

Data were prospectively recorded in the program SPSS 15.0 for Windows, also used for statistical analysis.

The categorical variables were expressed as frequency, being compared by use of chi-square test. The continuous variables were expressed as mean and standard deviation or median and interquartile range, according to their distribution, and compared by use of paired Student *t* test or Mann Whitney U test. The baseline values and those at 3, 6, 9 and 12 months from Doppler echocardiography were compared by use of one-way analysis of variance (ANOVA).

Cox regression analysis was used to identify independent echocardiographic variables predictive of cardiotoxicity.

Receiver operating characteristic (ROC) curves were created to define the most accurate cutoff points for the continuous variables independently associated with the event assessed.

The intra- and interobserver variabilities were analyzed with intraclass correlation coefficients, and Bland-Altman plots were created to show the results of the interobserver analyses.

For all tests, the statistical significance level adopted was p ≤ 0.05.

## Results

Of the 58 female patients consecutively referred to the Oncology Outpatient Clinic of the HUCFF, 49 were included in this study. Nine patients were excluded because of their high body mass index (BMI), which generates an inappropriate acoustic window to the LV GLS acquisition and EF calculation with the Simpson's method.

The mean age of the population studied was 49.7 ± 12.2 years, and the follow-up duration, 381 ± 29,8 days. Table 1 shows the baseline characteristics of the patients included in this study and of those excluded from it.

**Table 1:** General characteristics of the population included in the study and excluded from it.

Variable	population included n= 49	population excluded n = 9	p
Age (years)^* ll^	49.7 ± 12.2	51.0 ± 12.9	0.78
Weight (kg)^* ll^	67.6 ± 12.6	90.5 ± 12.5	< 0.05
Height (m)^* ll^	1.5 ± 0.06	1.5 ± 0.09	0.75
BSA (m^2^)^* ll^	1.65 ± 0.2	1.9 ± 0.2	< 0.05
BMI (kg/m^2^) †§	26.1 (23.6 - 30.4)	37.9 (31.6 - 40.9)	< 0.001
SBP (mm Hg)^* ll^	125.1 ± 17.4	132.2 ± 12.0	0.25
DBP (mm Hg)^* §^	74.7 ± 12.0	84.4 ± 5.3	0.02
HR (bpm)^* ll^	77.2 ± 10.1	83.4 ± 13.7	0.12
EF (Teicholz - %)^* ll^	69.0 ± 0.7	67.7 ± 9.3	0.59
Total Anthracycline Dose (Equivalence) (mg/m^2^)^†§^	600 (534-760)	600 (507-590)	0.68
Total Traztuzumab Dose (mg/m^2^)^* ll^	6823.3 ± 2395.6	7079 ± 2207.6	0.88
SAH ^‡^	16 (32.7)	4 (44.4)	0.37
Type II DM ^‡^	2 (4.1)	0	0.71
Beta-blocker^‡^	0	1 (11.1)	0.15
ACEI / ARB ^‡^	0	1 (11.1)	0.15
ASA ^‡^	2 (4.1)	0	0.71
HCTZ ^‡^	14 (28.6)	3 (33.3)	0.52
Statin ^‡^	3 (6.1)	0	0.59
Right breast CA ^‡^	24 (49.0)	8 (88.9)	0.03
Left breast CA ^‡^	25 (51.0)	2 (22.2)	0.11
Invasive ductal carcinoma ^‡^	34 (69.4)	9 (100)	
Lobular carcinoma ^‡^	7 (14.3)	0	0.16
Other subtypes ^‡^	8 (16.3)	0	
Pre-chemo surgery ^‡^	20 (40.8)	3 (33.3)	0.49
Radiotherapy ^‡^	26 (53.1)	7 (77.8)	0.16
Doxorubicin ^‡^	20 (40.8)	2 (22.2)	0.25
Epirubicin ^‡^	29 (59.2)	7 (77.8)	0.25
Trastuzumab ^‡^	8 (16.3)	2 (22.2)	0.48

* Mean (standard deviation);

† Median (25^th^ - 75^th^ Percentile);

‡ N (%); BSA: Body Surface Area; BMI: Body Mass Index; SBP: Systolic Blood Pressure; DBP: Diastolic Blood Pressure; HR: Heart Rate; EF: Ejection Fraction; SAH: Systemic Arterial Hypertension; DM: Diabetes Mellitus; ACEI: Angiotensin-Converting-Enzyme Inhibitor; ARB - Angiotensin Receptor Blocker; ASA: Acetylsalicylic Acid; HCTZ: Hydrochlorothiazide; CA: Cancer; Chemo: Chemotherapy; + Median (25^th^ - 75^th^ Percentile); bpm: beats per minute. Categorical variables compared by use of chi-square test ^‡^, p value ≤ 0.05. Continuous variables compared by use of Mann Whitney U test ^§^ or Student t test ^ll^, p value ≤ 0.05.

Regarding the oncological data, the most common histological type of tumor was invasive ductal carcinoma, observed in 70% of the patients. In 51% of the patients, the tumor was located in the left breast, 40.8% of the patients underwent surgery before chemotherapy, and 53.1%, radiotherapy (all of them after chemotherapy).

The patients underwent serial Doppler echocardiography, the first test being performed prior to treatment, and the following tests, on the third, sixth, ninth and twelfth months, in accordance to the study protocol. The LV GLS and the EF (Simpson's method) were obtained at all tests of the 49 patients. The mean time between undergoing the first Doppler echocardiography and initiating the antineoplastic treatment was 9 days.

The population studied and that excluded from the study were compared, and a similarity between the groups was observed.

### Intra- and interobserver analysis of global longitudinal strain

**Figure 1:**
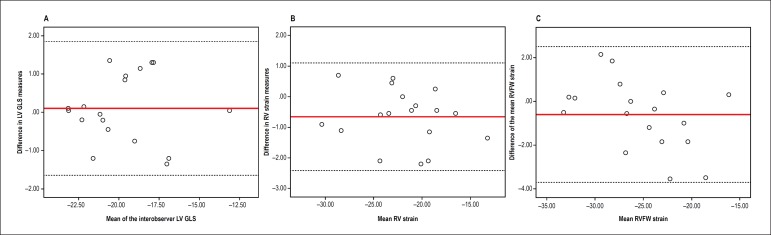
Bland-Altman plots showing interobserver analysis of left ventricular global longitudinal strain (LV GLS), right ventricular (RV) strain and right ventricular free wall (RVFW) strain in A, B and C, respectively.

The intraobserver intraclass correlation coefficients for LV GLS, RV strain, and RV free wall strain were 0.97 (95%CI: 0.91-0.99), 0.98 (95%CI: 0.93-0.99) and 0.98 (95%CI: 0.95-0.99), respectively. The interobserver intraclass correlation coefficients were 0.97 (95%CI: 0.92-0.99), 0.97 (95%CI: 0.92-0.99) and 0.98 (95%CI: 0.93-0.99), respectively. The results showed excellent inter- and intraobserver agreements. The excellent result of the interobserver analysis of the LV GLS, RV strain, and RV free wall strain can also be observed in Figures 1A, 1B and 1C (Bland-Altman plots).

### Characteristics of the population that developed cardiotoxicity

All patients in our study received anthracyclines, and 80% of them underwent radiotherapy after chemotherapy. During the follow-up, five patients (10%) developed cardiotoxicity, two on the third month and three on the sixth month. Despite the lack of a statistically significant association, the mean age of the patients with cardiotoxicity was higher than that of the 44 patients without it. In addition, 80% of those patients underwent radiotherapy, which is clinically relevant. All patients used anthracyclines. For two patients (40%) who developed cardiotoxicity, trastuzumab was associated to the antineoplastic regimen. The baseline characteristics of the patients who developed cardiotoxicity are shown in Table 2.

**Table 2:** Baseline characteristics of the patients treated with anthracyclines and trastuzumab - Association with cardiotoxicity.

Variable	Cardiotoxicity		P
	Yes	No	
	n = 5	n = 44	
Age (years)* ^ll^	56.4 ± 9.50	48.9 ± 12.30	0.60
Weight (kg)* ^ll^	65.8 ± 10.80	67.9 ± 12.90	0.78
Height (m)* ^ll^	1.58 ± 0.07	1.57 ± 0.08	0.80
BSA (m^2^)* ^ll^	1.63 ± 0.17	1.66 ± 1.17	0.80
BMI (kg/m^2^)^† §^	27.3 (22.9-29.2)	26 (23.7-30.4)	0.94
SAH^‡^	2 (40%)	14 (31.8%)	0.53
White ethnicity ^‡^	4 (80%)	27 (61.4%)	0.39
Mixed ethnicity ^‡^	1 (20%)	17 (38.6%)	
Type II DM ^‡^	1 (20%)	1 (2.3%)	0.20
ASA ^‡^	1 (20%)	1 (2.3%)	0.20
Diuretic ^‡^	2 (40%)	12 (27.3%)	0.45
Statin ^‡^	0	3 (6.8%)	0. 72
SBP (mm Hg)^‡ ll^	128 ± 23.9	124.8 ± 16.9	0.30
DBP (mm Hg)^‡ ll^	72 ± 13.0	75 ± 12.0	0.88
HR (bpm)^‡ ll^	78.4 ± 8.3	77.1 ± 10.4	0.78
Invasive ductal carcinoma ^‡^	3 (60%)	31 (71%)	
Lobular carcinoma ^‡^	2 (40%)	5 (11.4%)	0.17
Other types ^‡^	0	8 (18.2%)	
FEC protocol ^‡^	4 (80%)	27 (61.4%)	0.64
AC protocol ^‡^	1 (20%)	17 (38.6%)	
Radiotherapy ^‡^	4 (80%)	22 (50%)	0.35
Total Trastuzumab Dose (mg/m^2^)* ^ll^	4257 ± 1899	6692 ± 2352	0.24
Total Anthracycline Dose (Equivalent dose mg/m^2^)^† ll^	480 (402-720)	600 (525-795)	0.17

* Mean (standard deviation);

† Median (25^th^ - 75^th^ Percentile);

‡ N (%); BSA: Body Surface Area; BMI: Body Mass Index; SAH - Systemic Arterial Hypertension; DM: Diabetes Mellitus; ASA: Acetylsalicylic Acid; SBP: Systolic Blood Pressure; DBP: Diastolic Blood Pressure; HR: Heart Rate; FEC: 5-Fluorouracil + Epirubicin + Cyclophosphamide; AC: Doxorubicin + Cyclophosphamide. Categorical variables compared by use of chi-square test ^‡^, p value ≤ 0.05. Continuous variables compared by use of Mann Whitney U test ^§^ or Student t test ^ll^, p value ≤ 0.05.

### Description of the echocardiographic parameters

The means of the echocardiographic variables of the patients with and without cardiotoxicity are shown in Table 3. On the third month, the mean LV GLS, as well as its difference regarding the baseline value, were significantly higher in the group with cardiotoxicity. Although the EF value on the third month differed between the groups, its difference from the baseline value did not behave like that. On the sixth month, there was a significant drop in the EF and LV GLS, in addition to changes in the S wave of the left ventricle and E/E'.

**Table 3:** Echocardiographic characteristics of the patients treated with anthracyclines and trastuzumab - Association with cardiotoxicity.

ECHO	Variable*	Cardiotoxicity		P
		Sim	Não	
		n = 5	n = 44	
Baseline ECHO	EF (%)	64 ± 4.8	68.3 ± 7.7	0.120
	GLS (%)	-19.3 ± 1.2	-20.5 ± 2.0	0.100
	E/E'	8.9 ± 2.5	7.9 ± 1.6	0.450
	LV S (cm/s)	7.8 ± 1.1	8.3 ± 1.1	0.380
	RV S (cm/s)	12.6 ± 2.1	12.9 ± 2.0	0.760
ECHO 3 months	EF (%)	57.6 ± 12.3	67.2 ± 6.4	0.006
	EF Dif. 3 months (%)	6.4 ± 16.2	1.1 ± 7.2	0.190
	GLS (%)	-15.2 ± 2	-19.6 ± 2.1	0.005
	GLS Dif. 3 months (%)	4.1 ± 1.6	0.8 ± 1.6	0.008
	E/E'	7.1 ± 1.6	8.6 ± 1.9	0.230
	LV S (cm/s)	8 ± 0.8	8.5 ± 1.6	0.600
	RV S (cm/s)	12.6 ± 2.2	12.9 ± 2.3	0.800
ECHO 6 months	EF (%)	52 ± 5.1	67.4 ± 6.6	0.001
	EF Dif. 6 months (%)	12 ± 5.2	0.9 ± 9.8	0.004
	GLS (%)	-15.6 ± 1.1	-19.4 ± 2	< 0.001
	GLS Dif. 6 months (%)	3.7 ± 1.8	1 ± 1.6	0.026
	E/E'	9	8.2 ± 2.4	0.040
	LV S (cm/s)	6.3 ± 0.5	7.8 ± 1.4	< 0.001
	RV S (cm/s)	11.8 ± 1.6	13 ± 2	0.200

* Means ± SD; ECHO: Echocardiography; EF: Ejection Fraction (Simpson's); EF Dif.: Ejection Fraction Difference; GLS Dif.: Global Longitudinal Strain Difference; E/E': Ratio between E and E' wave values on Doppler echocardiography; LV S: S wave of the left ventricle; RV S: S wave of the right ventricle. The echocardiographic variables were compared by using paired Student t test, p value ≤ 0.05.

Table 4 shows the five cases of cardiotoxicity.

**Table 4:** Description of the cases with cardiotoxicity.

Cases	EF			LV GLS		
	Baseline	3 Months	6 Months	Baseline	3 Months (% ∆GLS)	6 Months
1	66%	52%	58%	-19.4%	-12.9% (33.50)	-17.0%
2	65%	69%	50%	-18.7%	-16.0% (14.40)	-14.3%
3	56%	69%	49%	-19.2%	-16.5% (14.10)	-15.5%
4	64%	58%	45%	-21.2%	-17.4% (17.90)	-15.0%
5	69%	40%	53%	-18.0%	-13.3% (26.10)	-16.4%

EF: Ejection Fraction (Simpson's method); GLS: Global Longitudinal Strain; % ∆GLS: Percentage Variation of Global Longitudinal Strain.

**Figure 2:**
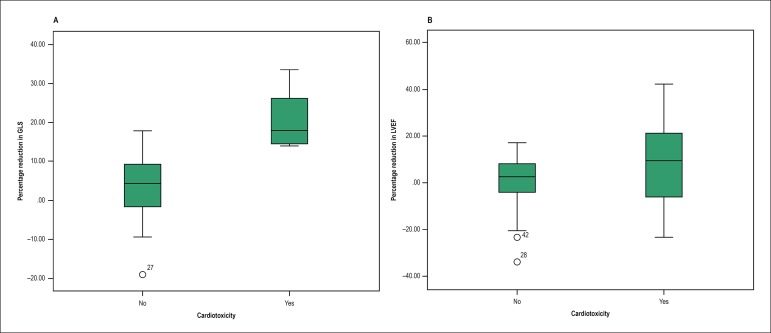
Boxplot illustrating the difference between the groups with and without cardiotoxicity. A, percentage reduction in left ventricular global longitudinal strain (GLS) variation; and B, percentage reduction in left ventricular ejection fraction (LVEF) variation.

When assessing the percentage reduction in the LV GLS, from baseline to the third month, between patients with and without cardiotoxicity, a clear difference is observed between the two groups (Figure 2A). That same behavior was not observed when assessing the percentage reduction in the EF in the same period, confirming that EF is not as sensitive as GLS to diagnose cardiotoxicity (Figure 2B).

The RV strain and RV free wall strain were acquired by using the same software developed for the analysis of the left ventricle, and showed mild non-significant changes on the third and sixth months, with subsequent normalization. However, TAPSE and tissue Doppler of the tricuspid annulus, measures related to the right ventricle, did not change during the follow-up.

### Predictors of cardiotoxicity

Aiming at assessing the association of each echocardiographic variable with cardiotoxicity (outcome), Cox regression analysis was performed (Table 5).

**Table 5:** Cox Regression Models.

	B	SE	p	HR	95%CI
**Cox regression model (Univariate)**					
Diastolic function	0.551	0.221	0.013	1.735	1.126-2.675
Left Atrial Volume (ml/m^2^)	- 0.354	0.154	0.022	0.702	0.519-0.950
LVEF (%)	- 0.117	0.046	0.011	0.889	0.813-0.973
GLS (%)	1.020	0.353	0.004	2.773	1.389-5.536
**Cox regression model (Multivariate - A)**					
Left Atrial Volume (ml/m^2^)	- 0.218	0.249	0.382	0.804	0.494-1.311
LVEF (%)	0.108	0.084	0.198	1.115	0.945-1.314
GLS (%)	1.41	0.686	0.040	4.097	1.068-15.716
**Cox regression model (Multivariate - B)**					
LVEF (%)	0.143	0.103	0.163	1.154	0.944-1.412
GLS (%)	1.975	0.952	0.038	7.207	1.115-46.573
Diastolic function	- 0.153	0.345	0.658	0.858	0.436-1.688

B: Coefficient; SE: Standard Error; HR: Hazard Ratio; CI: Confidence Interval; LVEF: Left Ventricular Ejection Fraction; GLS: Global Longitudinal Strain.

The variables with p ≤0.05 on Cox regression univariate analysis went to multivariate analysis of independent predictors of cardiotoxicity: EF (Simpson's method), LV GLS on the third month, left atrial volume, and diastolic function. Two models were created, separating the information of left atrial volume and diastolic function, because both variables express similar information, and can be interpreted in the concept of collinearity. Only LV GLS on the third month remained an independent predictor of cardiotoxicity, maintaining a statistically significant association in the multivariate models, even when the variables selected on univariate regression were tested two by two.

### ROC curves to predict cardiotoxicity by use of LV GLS

**Figure 3:**
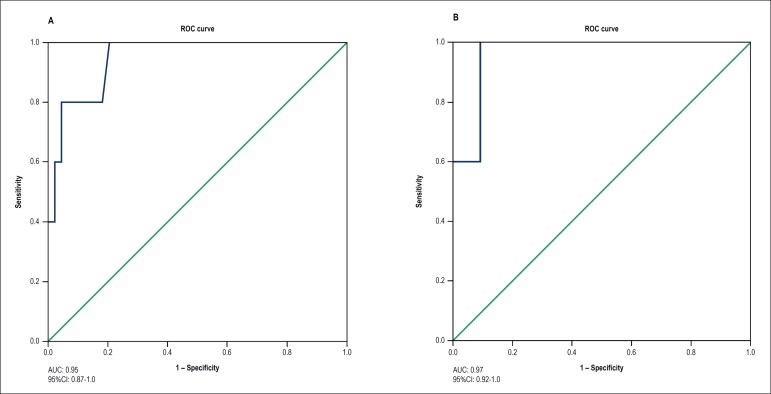
ROC curves to assess the cutoff point of the absolute value of left ventricular global longitudinal strain (GLS) (A), and the cutoff point of the percentage reduction in left ventricular GLS (B) as predictors of cardiotoxicity.

To define the most accurate cutoff point of the absolute LV GLS value on the third month to predict cardiotoxicity on the sixth month, a ROC curve was built (Figure 3A). The LV GLS value of -16.6 showed sensitivity of 80% and specificity of 95% to predict cardiotoxicity on the sixth month. Similarly, a second ROC curve was built to define the most accurate cutoff point of the percentage reduction of the LV GLS capable of predicting cardiotoxicity (Figure 3B). The LV GLS value of -14% showed sensitivity of 80% and specificity of 99% for that diagnosis. The accuracy of the percentage drop of 14% of the GLS (strain of the third month in regard to that of baseline) was assessed by use of its sensitivity and specificity (100% and 93%, respectively).

## Discussion

The results of the present study showed that the LV GLS was an excellent predictor of cardiotoxicity in our population, with high efficacy for its early diagnosis.

### Profile of morbidity of the population studied

Our population was considered to have a low morbidity profile. The incidence of the risk factors that could be related to cardiotoxicity was very low, and no statistically significant association could be demonstrated. That profile differs from that of other studies, which had cases of smoking, previous use of chemotherapy, radiotherapy, in addition to a higher frequency of systemic arterial hypertension and diabetes mellitus.^14,15^ The low morbidity profile can be associated with the lower incidence of cardiotoxicity observed in our population. Patients with the highest BMI were excluded from our study, because they could be considered at higher risk for cardiotoxicity, limiting the incidence rate of that event.

### Definition of cardiotoxicity

The definition of cardiotoxicity is fundamental, because it is not uniform in different studies, hindering the assessment of the real incidence of the event. The cardiotoxicity incidence in a systematic review published in 2014 ranged from 13% to 32%.^15^ Studies published by Sawaya et al.^16^ and Baratta et al.^17^ have found an incidence of 20%, using the same criterion of the trastuzumab committee. Our study found a cardiotoxicity incidence of 10%, lower than that reported by those studies. That could be explained by the low morbidity profile of our population, composed only by patients with breast cancer, with similar treatment protocols. In the study by Baratta et al.,^17^ if the cardiotoxicity incidence would be calculated only among patients with breast cancer, a 12% rate would be found, similar to that of our population.

### Characteristics of the population that developed cardiotoxicity

In our study, cardiotoxicity showed no statistically significant risk association with the clinical and anthropometric variables, histological type of tumor and treatment instituted. However, some variables evidenced clinically relevant information. The first was age, which was higher in the group that developed cardiotoxicity (mean of 56 years *versus* 49 years, in the group without cardiotoxicity), and could lead to a higher risk of events according to the literature. Another interesting variable, the total dose of anthracyclines and trastuzumab administered, which was lower in the group with cardiotoxicity, might be justified by the suspension or dose reduction of the antineoplastic drug by the oncology team in face of the drop in EF.

### Choosing the best time for doppler echocardiography

There is no consensus between the European and American Societies of Cardiology about the time during the treatment in which the echocardiographies should be performed. In our population, two patients had cardiotoxicity on the third month. Analyzing retrospectively, if the Doppler echocardiography would be performed after each anthracycline cycle, the drop in LV GLS might have occurred before the reduction in EF on the third month. Therefore, Doppler echocardiography would ideally be performed after the end of each anthracycline cycle.

### Marker of cardiotoxicity: 2D strain

Ejection fraction is not considered a good predictor of cardiotoxicity, because it does not detect early myocardial contractile function changes. Recent studies have demonstrated that strain changes precede EF changes in patients undergoing antineoplastic treatment.^14,16,18-21^ However, no consensus has been reached regarding the specific cutoff point of that variable that should be used as a predictor of cardiotoxicity.

The results of our study confirm LV GLS as an excellent independent predictor of cardiotoxicity, which can be assessed by use of the data from Cox regression (p = 0.004, HR = 2.77; 95%CI: 1.39-5.54). None of the patients assessed showed the LV GLS drop after the EF drop. The LV GLS change occurred from the third month onward, while EF (Simpson's method) changed only on the sixth month.

There is no consensus in the literature regarding the LV GLS value that can predict cardiotoxicity. Some articles have mentioned that, using the Speckle Tracking technique, a 10% to 15% reduction could predict that outcome. The last European recommendation from 2016 states that a reduction > 15% could predict cardiotoxicity, while a reduction < 8% could exclude its diagnosis. However, there is a grey zone between those values.^12,22^

Because of data inconsistency, our study aimed at finding the best cutoff point of the absolute value and percentage reduction of LV GLS to predict cardiotoxicity. The five events that occurred in our study enabled the construction of ROC curves to assess the diagnosis of cardiotoxicity on the sixth month. The need to define the ideal cutoff point of the GLS drop percentage capable of preventing cardiotoxicity has also been approached by some authors in recent years. According to Sawaya et al.,^16^ a 10% GLS drop on the third month of assessment could predict ventricular dysfunction occurring on the sixth month, with sensitivity of 78%, specificity of 79% and negative predictive value of 93%. The sample calculation of that study was based on the hypothesis that a 14% GLS drop could predict cardiotoxicity, exactly the same value found in our study.

It is worth noting that all prognostic models, in addition to predictive accuracy, should have the variables easily obtained. Doppler echocardiography is widely available and easily accessible, involves no radiation, being performed at the bedside. Its use in the follow-up of patients with breast cancer is a criterion of quality in healthcare services, mainly when using GLS, capable of predicting cardiotoxicity in those patients. However, it should be performed by echocardiography professionals trained in the method, with excellent image acquisition, to minimize the intra- and interobserver variabilities, using the same device and software, creating an individualized set for image acquisition and subsequent assessment. In our study, those values were found using the GE software. The different brands of devices have different normality range values. An agreement regarding those values has not been achieved between the manufacturers. Most studies and guidelines use a percentage variation of strain to define the presence of cardiotoxicity. Using the patient's baseline measures as control, and guaranteeing that all measures are taken with the same equipment and technique, the variations seem more reliable.

### Study limitations

The sequential echocardiographies were performed by the same examiner. Although the examiner was blind to the treatment instituted, an influence of the previous assessment on the subsequent tests could exist. However, the interobserver analysis showed an excellent correlation between data, and the second observer was blind not only to treatment, but also to the echocardiography times and previous results. Thus, although an assessment bias might have existed, it would not be strong enough to alter the results found.

The strain calculation requires an appropriate acoustic window. The patients excluded were those with the highest BMI, who would be at higher risk for cardiotoxicity according to the literature. In addition, in patients undergoing left breast surgery before the antineoplastic treatment, the presence of the expander or the surgical wound itself could interfere with the analysis. Limitations regarding the method also apply, and could be related to the test being performed by an untrained professional, or might be related to the devices available, taking into account that the strain values vary according to the brand of the device used.

Our study showed a low incidence of cardiotoxicity, which could limit the multivariate analysis. Currently, the literature is reviewing the assumption that a robust multivariate analysis should involve at least ten outcomes for each variable analyzed. There are three well-known simulation studies that assess that criterion for regression models, and they do not agree. Currently, in addition to the number of events per variable, the regression model depends on several other factors, such as the association of variables and outcomes, and some statistical studies report on the use of a smaller number of outcomes for each variable analyzed.^23,24^

## Conclusions

The incidence of cardiotoxicity associated with the antineoplastic treatment for breast cancer was 10% in our institution.

Our population with a low cardiovascular morbidity profile showed no association between cardiotoxicity and the risk factors classically described, such as clinical and anthropometric variables and treatment.

A significant LV GLS drop was observed from the third month onward, characterizing that variable as an independent predictor of cardiotoxicity, with a cutoff point of an absolute LV GLS value of -16.6% or a percentage LV GLS variation of -14%.
